# Using Image Processing in the Proposed Drowsiness Detection System Design

**Published:** 2018-09

**Authors:** Mohsen POURSADEGHIYAN, Adel MAZLOUMI, Gebraeil NASL SARAJI, Mohammad Mehdi BANESHI, Alireza KHAMMAR, Mohammad Hossein EBRAHIMI

**Affiliations:** 1. Research Center in Emergency and Disaster Health, University of Social Welfare and Rehabilitation Sciences, Tehran, Iran; 2. Psychosis Research Center, University of Social Welfare and Rehabilitation Sciences, Tehran, Iran; 3. Dept. of Occupational Health, School of Public Health, International Campus, Tehran University of Medical Sciences, Tehran, Iran; 4. Social Determinants of Health Research Center, Yasuj University of Medical Sciences, Yasuj, Iran; 5. Dept. of Occupational Health Engineering, School of Health, Zabol University of Medical Sciences, Zabol, Iran; 6. Occupational and Environmental Health Research Center, Shahroud University of Medical Sciences, Shahroud, Iran

**Keywords:** Driver drowsiness, Facial expression, Simulation driving, Road safety

## Abstract

**Background:**

Drowsiness is one of the underlying causes of driving accidents, which contribute, to many road fatalities annually. Although numerous methods have been developed to detect the level of drowsiness, techniques based on image processing are quicker and more accurate in comparison with the other methods. The aim of this study was to use image-processing techniques to detect the levels of drowsiness in a driving simulator.

**Methods:**

This study was conducted on five suburban drivers using a driving simulator based on virtual reality laboratory of Khaje-Nasir Toosi University of Technology in 2015 Tehran, Iran. The facial expressions, as well as location of the eyes, were detected by Violla-Jones algorithm. Criteria for detecting drivers’ levels of drowsiness by eyes tracking included eye blink duration blink frequency and PERCLOS that was used to confirm the results.

**Results:**

Eye closure duration and blink frequency have a direct ratio of drivers’ levels of drowsiness. The mean of squares of errors for data trained by the network and data into the network for testing, were 0.0623 and 0.0700, respectively. Meanwhile, the percentage of accuracy of detecting system was 93.

**Conclusion:**

The results showed several dynamic changes of the eyes during the periods of drowsiness. The present study proposes a fast and accurate method for detecting the levels of drivers’ drowsiness by considering the dynamic changes of the eyes.

## Introduction

Detecting the levels of drivers’ drowsiness has a key role in reducing the number of fatal injuries in traffic accident. Recent statistics and reports show that 20 to 50 million people are killed or injured in car crashes all over the world (1). Assessments conducted by US NHTSA (National Highway Traffic Safety Administration) showed that 100000 car accidents occur every year for which the drivers’ drowsiness is one of the principal contributor. These accidents cost over 12.5 billion$ and cause 1550 deaths and 71000 injuries (2). National Sleep Foundation of USA declared that 54% of adult drivers had driven during sleepiness and 28% of these drivers had fallen asleep completely (3). Road Safety Council of Germany (DVR) (Deutsche Verkeh Rswacht) states that 25% of fatal car crashes in highway traffic is due to momentary sleepiness (4). Investigations conducted by the Iranian legal medicine have shown that traffic accidents represent a large number of the country’s fatalities. According to the reports by this organization, such accidents cost over $4 billion annually that is 3.5 times gross domestic product of Iran. Based on the reports represented by police, approximately, 23% of car accidents in Iran was due to drowsy driving and driver fatigue (5, 6).

Having considered such statistics, devising the systems accurately to determine the levels of drivers’ drowsiness is of prime importance in order to reduce the number of car accidents. Generally, there three kinds of systems exist to determine the levels of drivers’ fatigue: techniques based on physiological signals, techniques based on driver’s performance and techniques based on image processing.

In methods developed based on physiological signals, some electrodes are attached to the body to detect the signals from brain and heart. This method would be irritating and is considered as a nuisance to the drivers (7). In methods developed based on the driver’s performance, much time is required to analyze driver’s performance, which consequently leads to low accuracy. In some cases, while the driver falls asleep for a moment, the status of the vehicle does not change, so, the system is disturbed in detecting micro-sleeps (8).

However, methods developed based on image processing are fast and precise to detect drivers’ drowsiness. Fatigue and drowsiness lead to some apparent signs on driver’s face. Interpreting such signals are the principals of the methods developed based on the image processing. In many cases, the first step in image processing is to recognize the facial zone from the received images.

Then, the parameters related to drowsiness such as eyes are recognized and their changes are investigated to detect the levels of sleepiness (9). By photographing the driver and image processing, the visual signals of sleepiness could be detected. Amongst various facial features, eyes are relatively of more importance and many studies have been conducted on the processing of the condition of the eyes. For instance, some factors like PERCLOS (Percentage of Eye Closure), duration of eyes closure and the number of blinks were utilized by IR Illuminator to determine the vigilance level (10). Drowsiness was detected merely based on PERCLOS. A small camera conducted the observing test detecting the levels of drowsiness by the number of eyes blinks and accuracy of 98% (11).

Regarding the importance of detecting the levels of drowsiness precisely without irritating the driver and considering the fact that almost no real time techniques for such purpose have been mentioned in the previous studies, we conducted an investigation of drowsiness by image processing of professional drivers and observed the movements of their eyes within the sequence of images.

## Materials and Methods

Current study was experimentally conducted among suburban male professional drivers using a driving simulator based on virtual reality laboratory of Khaje-Nasir Toosi University of Technology in 2015. The subjects were five average male drivers with normal appearance (with no abnormal facial hair, beard or mustache) who were randomly selected (12). They had no eyesight weakness (no glasses) whatsoever and had minimum two years of driving experience. Moreover, there was no record of persistent pain, use of contact lenses, musculoskeletal disorders such as low back pain and other diseases among the drivers (13,14). The main objective of this study was to determine the relative levels of drowsiness among the subjects, and not the total driving fatigue levels (5) as investigating the issues such as fatigue, entails a lot of effort and is highly complex (15,16).

### Preparation of the driving simulator

**Fig. 1:**
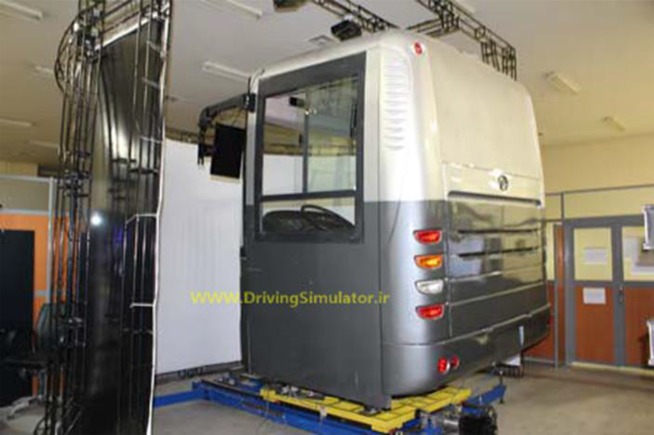
Driving simulator model AKIA-BI 301

In this research, in order to detect the levels of drowsiness and recording images from the drivers, virtual-reality driving simulator was utilized in a room where levels of illumination, noise, and temperature were controlled., simulator model AKIA-BI 301BI 301 Full was used to conducting the test (Fig. 1). The road was a highway with 3 lines, no side details, and the low meander to inspire drowsiness and designed for 2 h sessions (5).

### Protocol of testing

For initiating the test, the driver starts the simulator. As the test begins, camera records images of driver’s face. Meanwhile, observer interprets images to recognize the levels drowsiness. When simulator shows the road, crossing test finishes. (Four wheels should exit the road according to the researcher’s assessment). For controlling, the light from the vehicles ahead that caused bright light shock leading to reduced subjective sleepiness (17) the oncoming vehicles made use of low beam and the number of them was reduced. In order to control the circadian rhythm, the tests were performed between 9 A.M to 12 A.M (18, 12).

### Drowsiness detection model

**Fig. 2:**
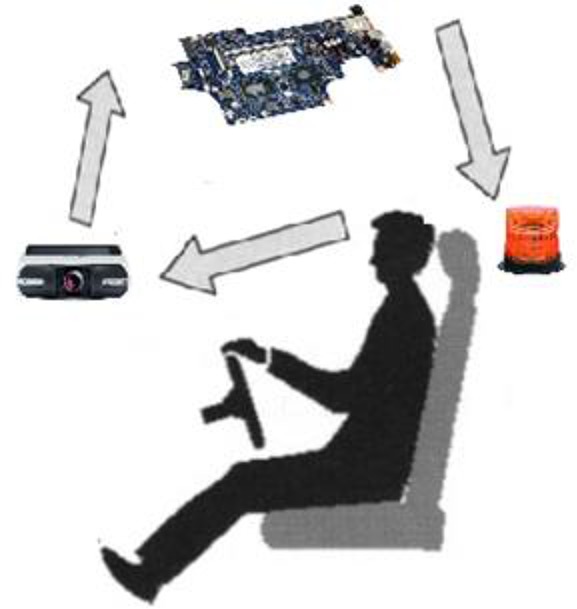
Schematic view of driver, camera and image processor

In this part of the research, we developed software that could receive color frames from the camera placed in front of the driver and calculate coordination of facial details. Finally, by comparing and fissuring, the information obtained from interpreting assessment criterion about the alertness or sleepiness together with information resulted from image processing, the software for detection of the levels of drowsiness was developed and promoted. This method is conducted in several steps as follows: first, the image of driver’s facial features taken by the camera is transferred to a processor to be processed (Fig. 2).

**Fig. 3:**
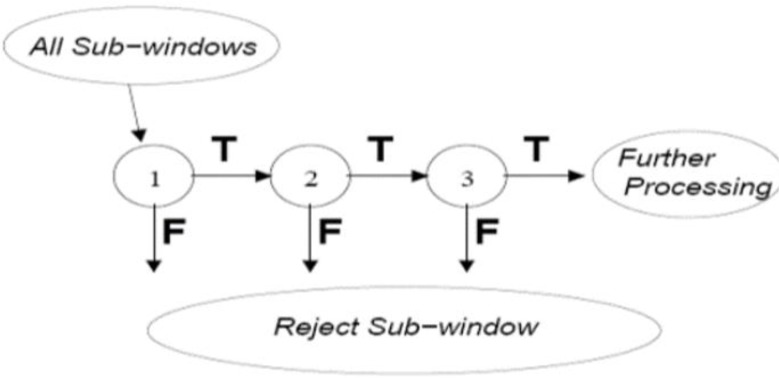
Categorizers are imposed in a cascade sequence

**Fig. 4:**
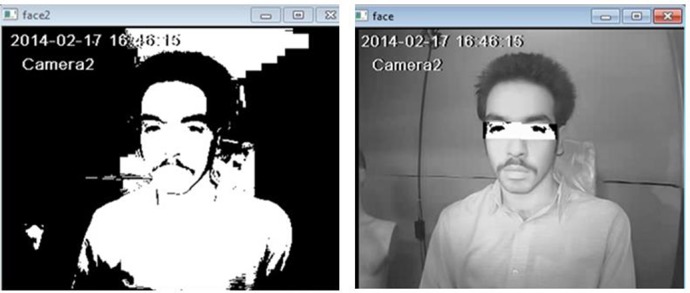
Eyes detection by Violla-Jones algorithm

Next, the facial features and location of the eyes are determined by Violla-Jones algorithm (19). For convenience, categorizers are utilized in the cascade sequence (Fig. 3). In this method, driver’s face was detected with regard to the oval shape of the head, hue and the eye sockets. Then, the zone of the eyes was recognized by considering the various changes in derivatives (between pupil and white part of eyes) of the visual information (Fig. 4).

**Fig. 5:**
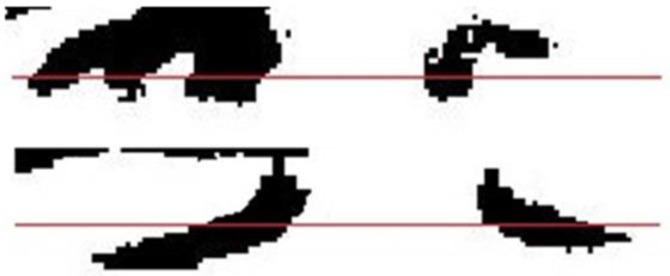
The ratio of black pixels in upper part of the image of eye to the black pixels in lower part while eyes are open (upper section) and closed (lower section)

Finally, to recognize whether eyes are closed or open, images are converted into gray scale format. Then, by calculating the mean of component V, illumination of images is normalized. Afterward, image of eyes is converted into binary by determining threshold via OTSU method. This conversion reduces the volume of data. After that, the image is divided into upper and lower part. When eyes are open, due to the color of pupils and eyelash, the ratio of dark pixels in upper part of eye is greater than the state of closed eyes because in the case of closed eyes, eyelids with light color cover pupils. In addition to this, in this state eyelash is in the lower part. To improve images, combining and extending wear were utilized to remove black spots. Then, the ration of black pixels to the whole pixels was calculated in both upper and lower parts. Finally, the ration of these amounts was used to recognize whether eyes are open or closed (Fig. 5) the procedure mentioned above: This figure is two sections for differences between open and closed eye.

## Results

**Fig. 6:**
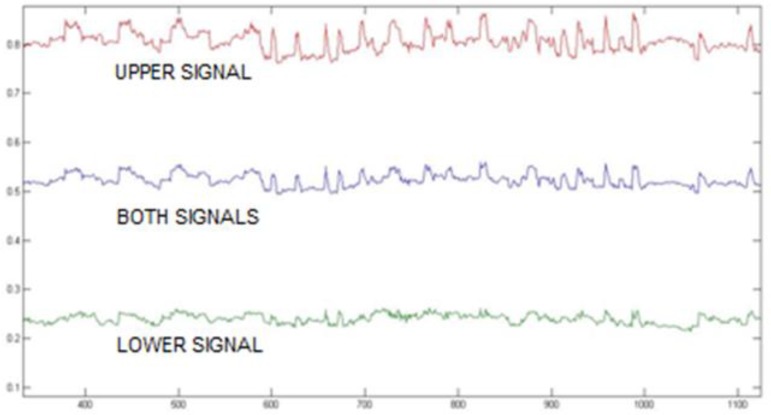
Changes in black and white pixels in upper and lower parts of the image in a time interval (during open and closed eyes and blink detection by these characteristics)

**Fig. 7:**
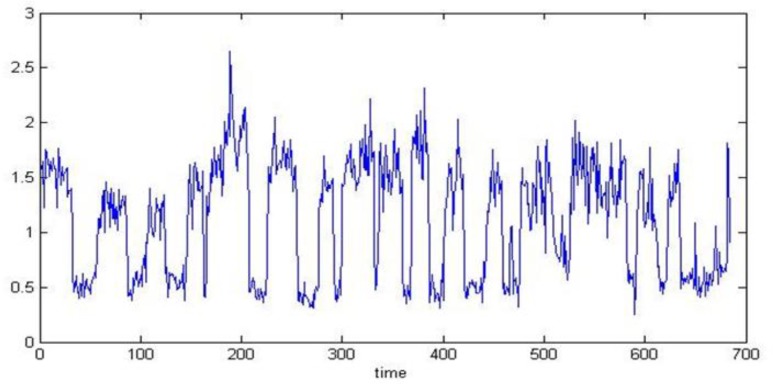
Changes in black and white pixels in upper and lower parts of the image in a time interval (scaled up by 3)

The results of analyzing frames of the eye processing are shown in Fig. 6 as changes in ratio of black and white pixels in upper and lower parts and compound signal when eyes are closed, open, and recognizing blinks by processing properties. To scale up important changes which are the basis of the upper signal for tracking, this property is multiplied by 3 to be illustrated more obvious in Fig. 6. Fig. 6 shows changes in black pixels in upper part of the image with respect to the lower part. The difference of this ratio of open and closed eyes is significant. By determining an appropriate threshold, open and closed eyes are detected. Mean of the lower part is appropriate to distinguish open and close eyelids (Fig. 7).

**Fig. 8:**
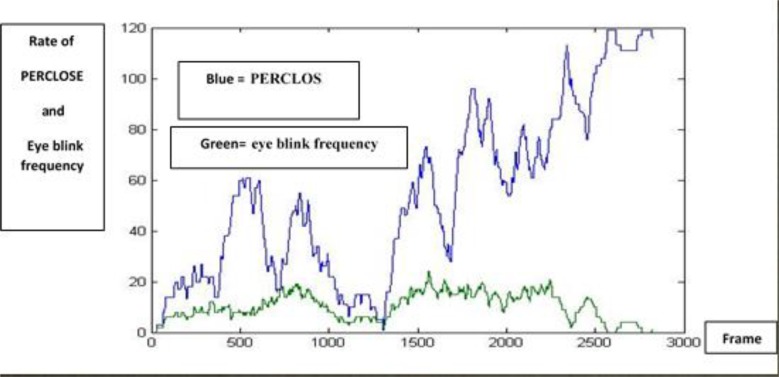
Rates of PERCLOS and eye blink frequency are shown by blue and green line graphs, respectively

After detecting closed or open eyes, blink duration (interval between open and closed eyes), blink frequency, the number of long blinks and their averages in 5 min were obtained (Fig. 8).

Finally, by finding information on closed or open eyes, blinks frequency and blink duration and by utilizing neural network, level of drowsiness was recognized.

### Results from neural network

**Fig. 9:**
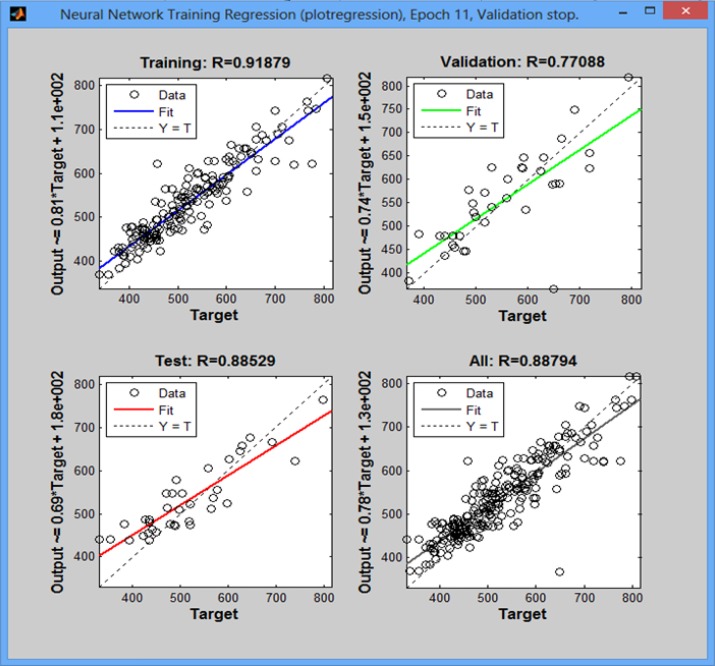
Regression of neural network pertained to a test to find appropriate neural network

To determine the levels of drowsiness by eyes signals, MLP neural network with three middle layers (with tansig function) and two inputs (first input: the averages of upper pixels and input: the averages of lower pixels) and one output (level of drowsiness) were utilized (Fig. 9). Full propagation and decreasing gradient methods were used. The number of training data was 9964 frames recorded from the five drivers’ sleepiness.

The network was taught by 1000 epoch. 70% of data and the rest of data (30% of data) for testing were transferred to the network for training.

The mean of squares of errors for data trained and was tested by the network were 0.0623 and 0.0700, respectively. Then, the level of accuracy was estimated at 93%.

## Discussion

Fatigue and drowsiness cause obvious changes in driver’s facial features and expressions and the position of head and eyes. Most of the studies conducted on the effects of fatigue and sleepiness have focused on the dynamic changes of the eyes and their movements during the periods that an individual is fatigued and sleepy. In this research, the level of drivers’ drowsiness was detected by employing an image-processing technique. In comparison with other techniques, this method detects the level drowsiness more accurately. For example, in the technique (20) that employs a color video camera placed directly in front of the driver, eyes are targeted to recognize micro-sleeps. In this research, we presented a method that in addition to the micro-sleeps, blinking (number and characteristics of blinks) is also monitored. While in another research (21) hue and Gabor filter were utilized, we considered the blink duration and frequency, which are relatively more advantages.

In a research conducted to detect sleepiness in 2008, the driver wore special glasses. A small camera was also placed on the glasses to monitor eyes their movements, only micro-sleep were detected, and blinks were considered quantitatively (22).

After detecting open and closed eyes, the criteria of blinking (like blink duration, blink frequency, open and closed eyelids); number of blinks during one minute, number of long blinks and their means in five minutes were calculated. Obviously, this research considers wider range of data and more criteria and processes more quickly.

In addition to the eyes, individuals’ posture, mouth, speech, and slumber are not normal during drowsiness. These were shown by the use of CCD camera in monitoring mouth features. They utilized FISCHER classifier to extract shape and place. In addition, by considering apparent geometrical characteristics of mouth they obtained a set of characteristics to detect one of three states of the mouth (23). Apparent changes of the mouth and eyes, changes in states of head and facial features are targeted to detect driver’s fatigue. For example, a system to detect small movements of head MINDS to recognize micro-sleeps and head dropping has been developed (24). In addition, a technique was introduced for detecting facial movements to recognize level of alertness (25).

In the future for detecting fatigue or sleepiness, other criteria like open or closed mouth, movements of head and facial features can be taken into account (23,25).

During conducting the tests on drivers by simulator and the software, appropriate illumination and location of the camera increased the efficacy of method (26). Besides, this method processes data more quickly and accurately than other methods. High rate of road fatality caused by driver fatigue highlight the importance of this method.

## Conclusion

A benefit of the method to detect drowsiness is intelligent sleepiness detection and use of various parameters in a long time and driving background. This advantage leads to detecting drowsiness in early stages and activate the alarm before a car accident occur. However, sleepiness is very complicated to be detected completely only by image processing. Therefore, various aspects of drowsiness were studied in virtual-reality driving simulator laboratory of Khaje Nasir University. Additionally, by using more criteria and obtained information by sensors drowsiness is detected.

## Ethical considerations

The approval of Tehran University of Medical Sciences was obtained for conducting the study. All participants were informed about the objectives of the study, and their informed consent was obtained.
